# Less common phenotypes of myelin oligodendrocyte glycoprotein antibody-related diseases in children deserve more attention

**DOI:** 10.1038/s41390-024-03058-x

**Published:** 2024-03-04

**Authors:** Xiao-yu Wang, Yan Jiang, Peng Wu, Jian-nan Ma, Ping Yuan, Xiu-juan Li, Li Jiang

**Affiliations:** Department of Neurology; Chongqing Key Laboratory of child Neurodevelopment and Cognitive Disorders; Ministry of Education Key Laboratory of Child Development and Disorders; National Clinical Research Center for Child Health and Disorders; China International Science and Technology Cooperation base of Child development and Critical Disorders, Children’s Hospital of Chongqing Medical University, Chongqing, PR China

## Abstract

**Background:**

To facilitate the identification of less common clinical phenotypes of myelin oligodendrocyte glycoprotein antibody-associated disease (MOGAD) in children.

**Methods:**

We retrospectively reviewed medical records of 236 patients with MOGAD. The following phenotypes were considered to be typical for MOGAD: ADEM, ON, TM, and NMOSD. Less common onset clinical phenotypes were screened out; their clinical and magnetic resonance imaging (MRI), diagnosis, treatment, and prognosis were summarized and analyzed.

**Results:**

16 cases (6.8%) presented as cortical encephalitis, with convulsions, headache, and fever as the main symptoms. 15 cases were misdiagnosed in the early period. 13 cases (5.5%) showed the overlapping syndrome of MOGAD and anti-N-methyl-D aspartate receptor encephalitis (MNOS), with seizures (92.3%) being the most common clinical symptom. 11 cases (84.6%) showed relapses. The cerebral leukodystrophy-like phenotype was present in seven cases (3.0%), with a recurrence rate of 50%. Isolated seizures without any findings on MRI phenotype was present in three cases (1.3%), with the only clinical symptom being seizures of focal origin. Three cases (1.3%) of aseptic meningitis phenotype presented with prolonged fever.

**Conclusion:**

40/236 (16.9%) of children with MOGAD had less common phenotypes. Less common clinical phenotypes of pediatric MOGAD are susceptible to misdiagnosis and deserve more attention.

**Impact:**

- This is the first comprehensive analysis and summary of all less commonl clinical phenotypes of MOGAD in children, while previous studies have only focused on a specific phenotype or case reports.
- We analyzed the characteristics of MOGAD in children and further revealed the reasons why these less common clinical phenotypes are prone to misdiagnosis and deserve more attention.
- Our research on treatment has shown that early detection of MOG antibodies and early treatment are of great significance for improving the prognosis of these patients.

## Introduction

The typical phenotypes of myelin oligodendrocyte glycoprotein antibody-associated disease (MOGAD) in children include acute disseminated encephalomyelitis (ADEM), optic neuritis (ON), neuromyelitis optica spectrum disorder (NMOSD), and transverse myelitis (TM), which comprise approximately over 90% of MOGAD phenotypes in children;^1^ these phenotypes are well known and becoming standardized in diagnosis and treatment. International MOGAD panel has proposed diagnostic criteria, in which the core demyelinating events are optic neuritis, myelitis, ADEM, cerebral monofocal or polyfocal deficits, brainstem or cerebellar deficits and cerebral cortical encephalitis often with seizures.^2^ The recent studies have revealed that approximately 10% of children gradually develop rare clinical phenotypes, such as cortical encephalitis, overlapping syndrome of MOGAD and anti-N-methyl-D aspartate receptor encephalitis (MNOS), cerebral leukodystrophy-like phenotype, isolated seizures without any findings on MRI, and aseptic meningitis, which further expand the spectrum of MOGAD.^3–8^

Shu et al. studied 35 patients with MOG-IgG-related cortical encephalitis and found that up to 42.9% of them were initially misdiagnosed as central nervous system infection and treated with anti-infective or anticonvulsant therapies, with an extremely poor outcome.^3^ Fan et al. found that 11.9% of children with MOGAD had MNOS.^4^ Zhang et al. showed that in pediatric anti-N-methyl-D-aspartate receptor (NMDAR) encephalitis cases, 16.9% of children were positive for myelin oligodendrocyte glycoprotein (MOG) antibodies and 20.2% had demyelinating lesions.^5^ In the study by Foiadelli et al. patients were not diagnosed as MOGAD due to the early onset of only seizures; 85.7% of the patients were thus treated with only antiepileptic medication, and 42.9% of the patients had poor control and relapse of seizures.^6^ Armangue et al. reported four cases of MOGAD with magnetic resonance imaging (MRI) findings similar to hereditary leukodystrophy.^7^ Gombolay et al. reported 10 patients with suspected meningitis with poor response to anti-infective therapy who were found to be positive for MOG antibodies; eight patients (80%) showed significant improvement in clinical symptoms with immunotherapy.^8^

Thus, studies on less common clinical phenotypes of pediatric MOGAD are scarce with most of them being case reports. Clinicians do not have in-depth insight into them, and there are plenty of misdiagnoses and mistreatments as the clinical features are unclear. Therefore, in this study, we retrospectively analyzed children with MOGAD for less common clinical phenotypes; their clinical and imaging features, diagnosis, treatment, and prognosis were summarized and analyzed to improve clinicians’ understanding of less common clinical phenotypes of MOGAD in children.

## Methods

### Patients

We retrospectively analyzed 236 children with MOGAD hospitalized in the Department of Neurology of Children’s Hospital of Chongqing Medical University, China, from January 2016 to April 2022.Typical for MOGAD were considered as the following phenotypes: ADEM, ON, TM, NMOSD. 40 cases with less common clinical phenotypes were identified. The following detailed clinical information of 40 children with less common clinical phenotypes of MOGAD was collected: 1. General information: name, sex, age of onset, diagnosis; 2. Clinical symptoms: antecedents, initial symptoms, clinical symptoms, and physical signs; 3. Objective information: serum and/or cerebrospinal fluid MOG-IgG antibody titer, complete blood count, serum C-reactive protein (CRP) level, erythrocyte sedimentation rate, serum procalcitonin (PCT) level, cerebrospinal fluid (CSF) examination, MRI, electroencephalogram; and 4. Treatment information: treatment during the hospitalization. The children were followed up for clinical recovery status, cranial MRI remission status, and presence or absence of disease relapse; annualized relapse rates were further measured.

### Antibody testing

Serum and/or cerebrospinal fluid MOG antibody and anti-NMDAR antibody were detected by fixed-cell-based assay. MOGAD was diagnosed by positive serum antibody, and anti-NMDAR encephalitis was diagnosed by positive cerebrospinal fluid antibody. Antibody titers of ≥1:10 were considered positive.

### Statistical methodology

SPSS 26.0 was used for analysis. Normally distributed data were described as mean ± standard deviation, and non-normally distributed data were expressed as median (range). Count data were expressed as number of cases and percentage (%).

## Results

**Fig. 1:**
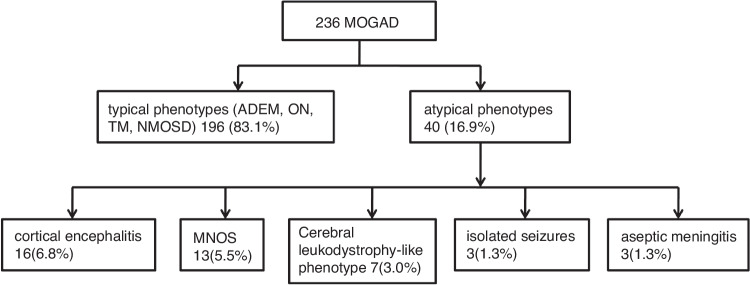
Case screening flow chart. Two hundred and thirty-six children with MOGAD were analyzed, of which 40 cases had less common clinical phenotypes, including 16 cases of cortical encephalitis, 13 of MNOS, 7 of cerebral leukodystrophy-like phenotype, 3 of isolated seizures without any findings on MRI, and 3 of aseptic meningitis.

We retrospectively analyzed 236 children with MOGAD, of which 196 cases (83.1%) had typical phenotypes, including ADEM, ON, TM, and NMOSD, and 40 cases (16.9%) had less common clinical phenotypes, including 16 cases of (6.8%) cortical encephalitis, 13 of (5.5%) MNOS, 7 of (3.0%) cerebral leukodystrophy-like phenotype, 3 of (1.3%) isolated seizures without any findings on MRI, and 3 of (1.3%) aseptic meningitis. Among these, two cases of cortical encephalitis overlapped with MNOS (Fig. 1).

### Cortical encephalitis

The clinical features, imaging characteristics, and treatment details of 16 children with cortical encephalitis phenotype are shown in Table 1. A total of 18 episodes of illness were observed in these children, with two patients experiencing two episodes. Seizures, the most commonly observed clinical symptoms, were seen in 16 episodes (88.9%). There were 12 (75%) bilateral tonic–clonic seizures of focal origin and 9(52.3%) status epilepticus. Other symptoms were headache in 13 (72.2%), fever in 11 (61.1%), dyskinesia in 7 (38.8%), hemiparesis in 5 (27.8%), speech disorder in 5 (27.8%), and cognitive deficits in 5 (27.8%). MOG antibodies were positive in 12 episodes, which were negative or undetectable at the time of the first episode in six cases. The CSF leukocytes fluctuated from 4 to 136 ×10^^6^/L, with 11 cases (61.1%) having a mononuclear cell count of >15 ×10^^6^/L. Six episodes (33.3%) had increased CSF protein levels (0.48–2.81 g/L). Cranial MRI of these children showed cortical hyperintensities on T2-weighted fluid-attenuated inversion recovery sequences. Six cases (33.3%) had sulcus or periaqueductal enhancement; 11 cases (61.1%) had unilateral cortical abnormalities and seven (38.9%) had bilateral cortical abnormalities. The frontal lobe was the most common site to be affected (66.7%), followed by the temporal lobe (38.9%), parietal lobe (33.3%), and occipital lobe (22.2%).

**Table 1:** Clinical features, imaging characteristics, and treatment details of children with cortical encephalitis (*n* = 16).

No.	Age(years) /Sex	Fever	Headache	Convulsive seizure		Decreased consciousness	Dyskinesia	Cognitive disorder	Language Disorders	MOG antibody titer			High T2FLAIR signal	Cerebral sulcus and/or meningeal enhancement	Treatment
										Serum		CSF			
1	4.6/F	−	−	+		+	−	−	+	1: 10		1:10	Bilateral	−	Anti-in, IVIg+HIMP
2	9.8/F	+	+	+		−	−	−	−	NT^a^		NT	Bilateral	+	Anti-in, anti-ep
3	7.3/M	+	+	+		−	+	−	−	1:100		1:320	Bilateral	+	Anti-in, IVIg
4	4.9/M	+	+	+		−	−	−	−	NT^a^		NT	Bilateral	−	Anti−in
5	8.5/M	+	+	+		−	−	−	−	1:32		1:100	Unilateral	−	Anti-in, IVIg+HIMP
6	10.8/M	+	+	+		−	−	−	−	1:100		1:10	Unilateral	−	Anti-in, anti-ep, IVIg
7	10.2/F	+	+	+		+	−	−	−	NT^a^		NT	Unilateral	−	Anti-in
8	2.8/F	+	−	+		−	+	−	−	1:10		−	Unilateral	−	Anti-in, anti-ep, IVIg+HIMP
9	12.3/M	+	+	+		+	−	+	−	NT^a^		NT	Bilateral	−	Anti-in
10	6.6/F	+	+	+		+	+	−	−	1:100		−	Unilateral	+	Anti-in, anti-ep, IVIg+HIMP
11 (1)	10.3/F	−	+	+		−	+	+	−	NT^a^		NT	Unilateral	+	antiviral
11 (2)		−	+	+		−	+	+	−	1:100		1:32	Unilateral	+	IVIg+HIMP
12	12.8/M	+	−	+		−	−	−	−	1:320		1: 10	Unilateral	−	Anti-in, IVIg
13	6.7/F	−	−	+		−	−	−	+	NT^a^		NT	Unilateral	+	Anti-in, anti-ep
14	6.0/F	−	+	+		+	−	−	+	1:100		1:10	Bilateral	−	Anti-in, IVIg
15	10.1/M	+	+	+		+	+	+	+	1:10		1:32	Bilateral	−	Anti-in, IVIg+HIMP
16 (1)	12.5/M	−	+	−		+	+	−	+	1:32		NT	Unilatera	−	Anti-in, refuse immunotherapy
16 (2)		−	−	−		−	−	+	−	1:32		1: 10	Unilate	−	Anti-in

Age: + yes, − no, *F* female, *M* male, *CSF* cerebrospinal fluid, *NT* not tested, *Anti-in* Anti-infection, *anti-ep* anti-epileptic, *IVIg* Intravenous immunoglobulins, *HIMP* High doses of intravenous methylprednisolon.

^a^Six patients were missing MOG antibody results because they did not test at other hospitals during their first episode or their parents were unwilling to test. However, when a demyelinating event occurred again, they tested positive for MOG antibodies. Based on the clinical manifestations and MRI, a retrospective analysis was conducted to determine the type of first episode as cortical encephalitis.

As shown in Table 2, 15 children (93.8%) were misdiagnosed at the disease onset, of which 10 (66.6%) were misdiagnosed with viral encephalitis, 3 (20.0%) with epilepsy, 1 (6.7%) with purulent meningitis, and 1 (6.7%) with stroke. At the initial diagnosis, these 15 cases received anti-infective therapy and five of them received antiepileptic therapy, with no improvement after 9 (interquartile range, IQR: 5–20) days of treatment. Nine cases received immunotherapy and showed significant improvement after 2 (IQR: 1–3) days of therapy. Three children without immunotherapy were discharged with the diagnosis of viral encephalitis; however, during follow-up, two of them developed ADEM and one developed MNOS, which later improved after receiving immunotherapy. Two cases were initially hospitalized with the diagnosis of epilepsy, and their symptoms improved after antiepileptic therapy before discharge. At a follow-up of 48 (IQR: 36–55) months, 11 cases (68.8%) relapsed after 1–11 months following the first episode of cortical encephalitis, with a total of 19 relapses. 4 (44.4%) of the 9 children treated with immunotherapy after the first attack had a relapse after 17.5 (IQR: 9.5–23.3) months ; the other 7 children who were not treated with immunotherapy had a relapse 1 (IQR: 1–2) month after the first attack, with eight episodes of (42.1%) ON, six of (31.6%) ADEM, two of (10. 5%) MNOS, and three of (15.8%) cortical encephalitis.

**Table 2:** Diagnosis and recurrence of episodes of children with cortical encephalitis.

No.	Initial diagnosis	Diagnosis on discharge	2^nd^ relapse	3^rd^ relapse	4^th^ relapse
1	Viral encephalitis	MOGAD (Cortical encephalitis)			
2	Viral encephalitis	Viral encephalitis	ADEM	ADEM	
3	Viral encephalitis	MOGAD (Cortical encephalitis))			
4	Viral encephalitis	Viral encephalitis	ADEM		
5	Viral encephalitis	MOGAD (Cortical encephalitis)	ON	ON	
6	Viral encephalitis	MOGAD (Cortical encephalitis)			
7	Viral encephalitis (with previous ON)	Central nervous system demyelinating disease (Cortical encephalitis)	Cortical encephalitis	ON	ON
8	Viral encephalitis	MOGAD (Cortical encephalitis)			
9	Viral encephalitis	Viral encephalitis	MNOS		
10	Viral encephalitis	MOGAD (Cortical encephalitis)	ON	ON	
11	Epilepsy	Epilepsy	Cortical encephalitis	ADEM	
12	Epilepsy	MOGAD (Cortical encephalitis)			
13	Epilepsy	Epilepsy	ON	MNOS	
14	Central nervous system demyelinating disease	MOGAD (Cortical encephalitis)	ADEM		
15	Purulent meningitis	MOGAD (Cortical encephalitis)	ADEM		
16	Stroke	MOGAD (Cortical encephalitis)	Cortical encephalitis	ON	

*ADEM* acute disseminated encephalomyelitis, *ON* optic neuritis, *MNOS* overlapping syndrome of MOGAD and anti-N-methyl-D aspartate receptor encephalitis.

### MNOS

**Fig. 2:**
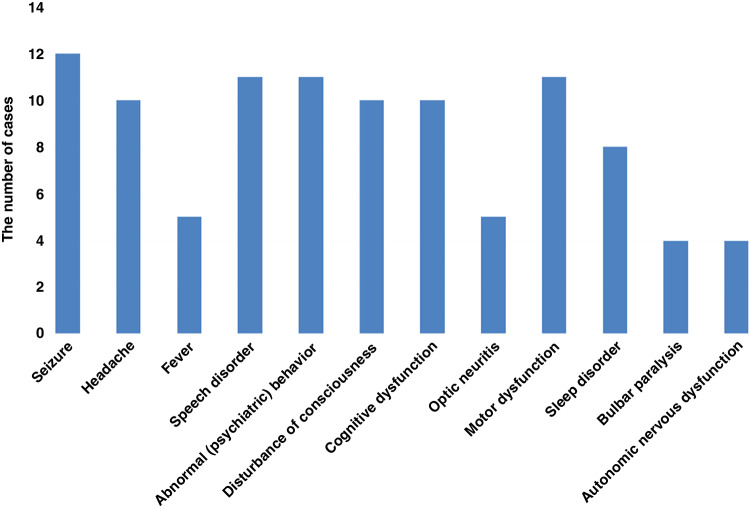
Clinical symptoms of 13 MNOS cases. The most common clinical symptoms were seizures (12/13), followed by abnormal (psychiatric) behavior (11/13), speech disorders (11/13), motor dysfunction (11/13), cognitive dysfunction (10/13), disturbance of consciousness (10/13), sleep disorders (8/13).

As shown in Fig. 2, 13 children with MNOS had a total of 30 acute episodes. At first attack, the most common clinical symptoms were seizures (92.3%), followed by mental-behavioral abnormalities (84.6%), language disorders (84.6%), motor disorders (84.6%), cognitive disorders (76.9%), consciousness disorders (76.9%), sleep disorders (61.5%), and visual disorders (38.5%). At the time of first onset, MNOS was diagnosed in seven (53.8%), MOGAD in four (30.8%), and NMDAR in two (15.4%) cases. Moreover, 10 of the 30 acute episodes who had symptoms of both MOGAD and anti-NMDAR encephalitis were positive for both blood MOG antibodies and CSF anti-NMDAR, seven (23.3%) showed as anti-NMDAR encephalitis symptoms only (such as abnormal psychiatric behavior, sleep disorder and cognitive dysfunction), and nine (30%) showed as MOGAD symptoms only(such as visual impairment, headache and consciousness disturbance). Furthermore, 13 children had supratentorial lesions on MRI (100%), with involvement of the subcortical white matter in 11 (84.6%), cortical gray matter in 8 (61.5%), basal ganglia in 7 (53.8%), and thalamus in 5 (38.5%) episodes. Moreover, infratentorial lesions were observed in eight cases (61.5%), including five (38.5%) in the cerebellum and four (30.8%) in the brainstem.

As shown in Table 3, with a median follow-up of 43 (IQR: 27–49) months, 11 cases (84.6%) relapsed. On average, the first relapse occurred 4 (IQR: 3–9) months after the first disease onset, the second relapse occurred at 17.5 (IQR: 12.3–27.3) months after the first onset, and the third relapse occurred at 46 months after the first onset in one child. Seven children received first-line immunotherapy (intravenous methylprednisolone and/or intravenous immunoglobulins) after the first diagnosis of MNOS, and five children (71.4%) relapsed by the last follow-up, with an annual relapse rate of 0.5 (IQR: 0.33–1). After the first relapse, 10 children received maintenance immunotherapy (five received mycophenolate mofetil, five received rituximab, one received regular intravenous immunoglobulin, and one received azathioprine), and three children (30%) relapsed by the last follow-up, with an annual relapse rate of 0.5 (IQR: 0.4–0.5), which was lower than the annual relapse rate of children not receiving immunotherapy (median 2.4, IQR: 2–2.9).

**Table 3:** Maintenance immunosuppressant treatment and annual relapse rate in children with MNOS.

No	Immunos Uppressant	Duration of immunosuppressant use		Relapse rate per Year before immunosuppressant use	Annual relapse Rate after immunosuppressant use	Maintenance Time (month)	Annual Relapse rate
		Number of episodes	Time since the first onset (month)				
1	MFM	2	5	2.4	0	14	0.2
2	RTX	2	3	4	0		0.25
3	RTX	2	4	3	0.3	14	0.5
4	MFM	3	12	2	0.5		0.75
5	RTX and MFM mycophenolate mofetil	3	10	2.4	0		0.5
6	AZA	2	24	0.5	0	19	0.2
7	MFM	2	3	4	0	35	0.25
8	/	/	/	/	/	/	0.33
9	/	/	/	/	/	/	/
10	RTX intravenous immunoglobulin	2	40	0.3	0.5	12	0.57
11	MFM	2	5	2.4	0	12	0.44
12	/	/	/	/	/	/	/
13	RTX	2	6	2	0	5	1

Note: Case 8 relapsed without immunosuppressant; cases 9 and 12 did not show relapse.

*MFM* mycophenolate mofetil, *RTX* rituximab, *AZA* azathioprine.

### Cerebral leukodystrophy-like phenotype

**Fig. 3:**
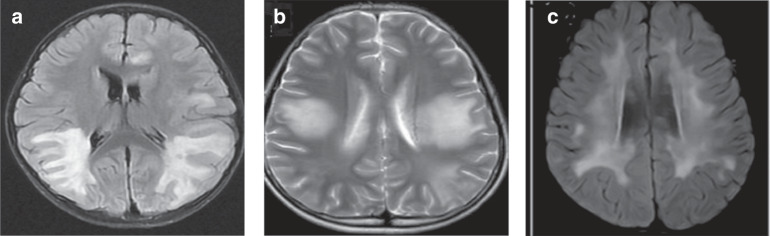
MRI features of cerebral leukodystrophy-like phenotype. **a** Paraventricular symmetric white matter lesions. **b** Large supratentorial symmetric cerebral white matter lesions. **c** Diffuse subcortical white matter changes.

Seven cases of cerebral leukodystrophy-like phenotype were noted, with a median age of onset of 5.3 (IQR: 4.1–6.5) years. Four (57.1%) of the seven children had motor disorders (three had hemiplegia and one had ataxia), three had consciousness disturbance (42.9%), three had cognitive disorders (42.9%), three had seizures (42.9%), three had headaches (42.9%), two had a fever (28.6%), two had visual disturbances (28.6%), two had speech disturbances (28.6%), two had personality changes (28.6%), and one had facial palsy (14.3%). The early symptoms of six children met the diagnostic criteria for ADEM. All seven children were positive for MOG antibodies, with titers ranging from 1:10 to 1:100. The cranial MRIs of all seven children showed cerebral leukodystrophy foci, involving subcortical white matter in seven (100.0%), deep white matter in five (71.4%), basal ganglia in five (71.4%), optic nerves in three (42.9%), thalamus in two (28.6%), brainstem in two (28.6%), and cerebellum in two (28.6%) cases. Figure 3 shows MRI brain images of three of these cases.

These children received immunotherapy at a median duration of 15 (IVIg+HIMP, IQR: 11–22) days after disease onset, with clinical improvement and a median Expanded Disability Status Scale score of 3 (IQR: 1.5–4.5) and 1 (IQR: 0–1) in the acute phase and at the time of discharge, respectively. One case was lost to follow-up, and the remaining six children were followed for a median duration of 27 (IQR: 12–42) months, with three children (50.0%) developing relapses. By the last follow-up, two children (33.3%) had visual impairment, one (16.7%) had epilepsy, and one (16.7%) had cognitive impairment.

### Isolated seizures without any findings on MRI

Among three cases of isolated seizures without any findings on MRI, the only clinical symptom was epileptic seizures, except for one case that presented with transient dizziness and headache that resolved quickly. Two cases (66.7%) had focal motor seizures and one (33.3%) had focal motor seizures and focal secondary bilateral tonic–clonic seizures. Two cases had normal electroencephalograms, and one case had focal spike-like slow waves in the sleep phase. Three children had normal cranial MRIs. Epilepsy was diagnosed at the time of the first seizure in two cases and viral encephalitis at the time of the first seizure in one case. In one case, a serum MOG antibody of 1:10 was measured at the initial onset. Serum MOG antibody was not tested in two cases at initial seizure onset, and their MOG antibody titer was measured to be 1:100 at the time of the reoccurrence of the demyelinating event.

The follow-up lasted for 20–44 months ; two cases had demyelinating events within 2 months after discharge, both presenting as ADEM, which subsided following immunotherapy. Two cases were persistently seropositive for MOG antibodies, and MOG antibodies turned negative in one case.

### Aseptic meningitis

All three children with aseptic meningitis had a fever and showed signs of meningeal irritation. Two cases had a fever for more than 2 weeks and one case had a fever for 5 days, with a mean duration of fever of 15.7 days. At the disease onset, the white blood cell counts were increased (13.38–27.45 × 10^^9^/L), with neutrophils predominating, with a serum CRP level of >8 mg/L in one case and normal serum CRP level in the rest. The serum PCT level was normal in all three cases before and after immunotherapy. CSF leukocytes were increased in all cases, and the CSF protein level was elevated by 1.31 g/L in one case. After immunotherapy, the CSF leukocyte and protein level were significantly decreased in two cases, and one case did not recheck the CSF. The serum MOG antibody titers of all three children ranged from 1:100 to 1:320. Two children were initially diagnosed with purulent meningitis and treated with anti-infective therapy for 19 and 23 days, respectively; no improvement was observed. Their symptoms significantly improved after receiving immunotherapy for 2 days. One child was initially diagnosed with viral meningoencephalitis and treated with antiviral therapy for 8 days with the addition of intravenous immunoglobulin, which showed marked improvement.

At 19–57 months of follow-up, one child was lost to follow-up and the remaining two had complete clinical remission without recurrence. The MRI lesion completely resolved in one case.

Table 4 shows the clinical information of the above five phenotypes.

**Table 4:** Summary of clinical information.

	Cortical encephalitis	MNOS	Cerebral leukodystrophy -like phenotype	Isolated seizures	Aseptic meningitis
Median age, years	9.1	11	5.3	10.3	4.3
Clinical presentation					
Seizures	88.9	92.3	42.9	100	33.3
Headache	72.2	76.9	42.9	0	66.7
Fever	61.1	38.5	28.6	0	100
Motor disorder	38.9	84.6	57.1	0	33.3
Cognitive dysfunction	27.8	76.9	42.9	0	33.3
Abnormal (psychiatric) behavior	0	84.6	0	0	0
consciousness disturbance	38.9	76.9	42.9	0	66.7
Sleep disorder	0	61.5	0	0	0
CSF WBC increase	66.7	46.2	42.9	33.3	100
MRI	Cortical hyperintensities 100	Supratentorial 100 infratentorial 61.5	Subcortical white matter 100	Normal	Non-specificity 66.7 meningeal enhancement33.3
Misdiagnosis	Viral Encephalitis62.5 Epilepsy 18.8 Purulent meningitis6.2 Cerebral stroke 6.2	ON 15.4 CCE 15.4 NMADR-E 15.4	ADEM 85.7	Epilepsy 66.7 Viral Encephalitis 33.3	Purulent meningitis 66.7 Viral Encephalitis 33.3
Time to first immunotherapy Median, days	14 (IQR: 7.5–20)	15 (IQR: 8–22)	15 (IQR: 11–22)	/	19 (IQR: 8–23)
Type of immunotherapy given					
IVIg+HIMP	37.5	38.5	100	0	66.7
IVIg	18.8	7.7	0	0	33.3
HIMP	0	7.7	0	0	0
Relapse	68.8	84.6	50	66.7	0

Numbers are expressed as percentages.

*MNOS* overlapping syndrome of MOGAD and anti-N-methyl-D aspartate receptor encephalitis, *CSF* cerebrospinal fluid, *MRI* magnetic resonance imaging, *IVIg* Intravenous immunoglobulins, *HIMP* High doses of intravenous methylprednisolon.

## Discussion

A total of 236 children with pediatric MOGAD were enrolled in our study; 40 (16.9%) of them were less common clinical phenotypes. The incidence of cortical encephalitis was 6.8%, which is in line with that of Shu H’s findings.^4^ Epileptic seizures were the most common symptom (88.9%), mostly presenting as seizures of focal origin, and 52.3% presented with status epilepticus, which is consistent with the results of Budhram et al.^9^ This phenomenon has been noted in the literature as FLAMES (fluid attenuated inversion recovery (FLAIR) hyperintense cortical lesions in MOG associated Encephalitis with Seizures).^2^ However, in Cristina’s study,^10^ Headache (79%) has a higher incidence than seizures (13, 68%) in cortical encephalitis patients. In our study, all children had focal cortical T2-weighted fluid-attenuated inversion recovery sequences of high signal on cranial MRI, with the frontal lobe being the most common site, and 33.3% showed adjacent sulcus and/or leptomeningeal enhancement. The majority (61.1%) of the children had unilateral cortical lesions, which is consistent with the findings of Wang Y ^11^and Tian F et al.^12^ however, 38.9% of the children had bilateral cortical lesions. Jain K et al. suggested that bilateral cortical encephalitis and underlying meningeal inflammation indicate a wider range of lesions in the cortical encephalitis phenotype of MOGAD.^13^ In the present study, up to 93.8% of the children were misdiagnosed at the disease onset; of these children, 66.6% were misdiagnosed as viral encephalitis. No improvement was observed after 5–20 days of treatment in 60% of these children; however, they showed significant clinical symptom improvement after 1–3 days of immunotherapy. Therefore, when there are clinical symptoms similar to viral encephalitis, cortical lesions on cranial MRI, and poor therapeutic effect, MOG antibodies should be tested as soon as possible to make an early diagnosis and use immunotherapy promptly.

A recent meta-analysis showed that approximately 9% of patients positive for MOG antibody also had positive NMDAR antibodies, and 7% of patients with anti-NMDAR encephalitis were MOG-IgG positive.^14^ In our study, 13 children with MNOS accounted for 5.5% of all MOGAD cases. In MNOS, 38.5%–57.1% of them had symptoms of anti-NMDAR encephalitis and MOGAD at the same time.^14,15^ Similarly, in our study, MOGAD and anti-NMDAR encephalitis was simultaneously observed in 53.8% of first episode. Seizures were the most common clinical symptom (92.3%), followed by mental-behavioral abnormalities, language disorders, and movement disorders. MNOS is more likely to present with seizures, mental-behavioral abnormalities, and language and movement disorders, which is in contrast to the study of Li^16^ et al. which reported that children with pediatric MOGAD present with encephalopathy, movement disorders, and visual disturbances as the most common symptoms. All 13 children with MNOS had supratentorial lesions on cranial MRI, predominantly in the subcortical white matter, cortical gray matter, basal ganglia, and thalamus, and more than half of these 13 children had infratentorial lesions in the cerebellum and brainstem. The cranial MRI abnormality rate of these children was much higher than that of children with anti-NMDAR encephalitis, as reported by Zhang et al. (10%–33%),^5^ and there was also a difference in the lesions sites because anti-NMDAR encephalitis often involves the limbic system; thus, the MRI abnormality rate (91.5%) and lesion site of children with MNOS are more in line with those of children with MOGAD reported by Li et al.^16^ Therefore, we believe that when children with MOGAD present with convulsions, mental–behavioral abnormalities, and language disorders, or when children with anti-NMDAR encephalitis present with significant cranial MRI abnormalities, particularly in the subcortical white matter and infratentorial lesions, MNOS should be highly considered. Interestingly, in our study, three children with only clinical symptoms of anti-NMDAR encephalitis had positive MOG antibodies, and four children with only clinical symptoms of MOGAD had positive CSF anti-NMDAR antibodies. Therefore, for the clinical consideration of MOGAD or anti-NMDAR encephalitis, simultaneous testing of MOG-IgG and anti-NMDAR-IgG is necessary. As per our findings, MNOS is more likely to recur than MOGAD and anti-NMDAR encephalitis alone and needs more aggressive immunotherapy.

Numerous studies have reported a cerebral leukodystrophy-like phenotype in MOGAD.^17–19^ In the present study, there were seven (4.7%) children with a cerebral leukodystrophy-like phenotype. Six children had an early clinical phenotype that met the diagnostic criteria for ADEM, which is consistent with the findings of a previous study that the cerebral leukodystrophy-like phenotype of MOGAD is a more symmetrical, widely fused white matter lesion of ADEM on MRI that develops over time.^17–19^ On average, the children in the present study received immunotherapy 15 days after the disease onset and showed good clinical improvement, suggesting that this phenotype of MOGAD is prone to delayed diagnosis and treatment. By the final follow-up, the relapse rate was 50%, with one case having three episodes of relapses, 33.3% of the children having visual impairment, and 16.7% still having residual epilepsy and cognitive impairment. This is similar to the findings of Hacohen and Armangue et al.^7,17^, in which the condition of all children improved clinically after acute treatment with steroids, but they were overall prone to relapse and had a poor prognosis.

Furthermore, 21.4–24.5% of pediatric MOGAD cases have seizures, which are common in ADEM, cortical encephalitis, and isolated seizures without any findings on MRI.^20,21^ isolated seizures without any findings on MRI account for approximately 20% of MOGAD cases with seizures.^20^ In the present study, three (1.3%) children were isolated seizure phenotype. All of them had focal motor or focal secondary bilateral tonic–clonic seizures, and one of them presented with status epilepticus. There were no specific changes in the electroencephalogram of all three children, which is in line with the findings of Ramanathan et al.^22^ Therefore, when clinically isolated seizures without any findings on MRI occur, particularly with focal motor seizures, and there is no structural abnormality on MRI and no specific abnormality on electroencephalogram, it is important to consider the isolated seizure phenotype of MOGAD.

Leinert et al.^23^ first reported aseptic meningitis as one of the clinical phenotypes of MOGAD in children. Vibha et al.^24^ reported a case of aseptic meningitis with MOGAD showing pia mater enhancement on MRI. In the present study, the first symptom of all three children with aseptic meningitis was recurrent fever with an average duration of up to 15.7 days, which is consistent with the clinical symptoms of meningitis. The results of blood routine examination showed a significant difference from the common bacterial infection. Therefore, we suggest that when meningitis is considered clinically, the leukocyte and neutrophil counts are elevated, but the serum PCT and CRP levels are not increased correspondingly, there is no evidence of pathogenesis, and anti-infective treatment is ineffective, the possibility of MOGAD should be considered.

There are other rare phenotypes of MOGAD described in the literature, such as intracranial hypertension^25^ and combined central and peripheral demyelination.^26,27^ In this study, we did not include these phenotypes because in our study there was only one case of each of those phenotypes. This may be related to insufficient number of cases. We will continue to collect cases and hope to make up for this deficiency in the future.

## Conclusion

Various less common clinical phenotypes of MOGAD have their own characteristics, and clinicians do not have enough understanding of them, which often leads to the misdiagnosis and mistreatment. Improving the understanding of them, early detection of MOG antibodies, early diagnosis, and active immunotherapy are of great significance for improving the therapeutic efficacy and prognosis of MOGAD in children.

## Data Availability

The datasets generated during and/or analyzed during the current study are available from the corresponding author on reasonable request.
